# Supplementary material for: Ultrasonic Cigarettes: Chemicals and Cytotoxicity are Similar to Heated-Coil Pod-Style Electronic Cigarettes
Source: Chem Res Toxicol. 2024 Jul 25;37(8):1329–43. doi: 10.1021/acs.chemrestox.4c00085 (PMC11337213; doi:10.1021/acs.chemrestox.4c00085)
Supplement: Supplementary file 1 — tx4c00085_si_001.pdf [file tx4c00085_si_001.pdf]

SUPPORTING INFORMATION

Ultrasonic Cigarettes: Chemicals and Cytotoxicity are Similar to Heated-Coil Pod-Style Electronic Cigarettes

Esther E. Omaiye[†], *Wentai Luo*[‡§], *Kevin J. McWhirter*[‡], *and Prue Talbot*[†*]

[†]Department of Molecular, Cell, and Systems Biology. University of California, Riverside, California 92521, USA

[‡]Department of Civil and Environmental Engineering, Portland State University, Portland, Oregon 97207, USA

[§]Department of Chemistry, Portland State University. Portland, Oregon 97207, USA

Corresponding Author
*Email: talbot@ucr.edu

TABLE OF CONTENTS

The Supporting Information is available free of charge.

PAGE S3 List of target aldehydes and their limits of quantification

PAGE S4 Heat map of flavor chemicals above the LOQ with concentrations below 1 mg/mL

PAGE S5 Flavor chemicals detected below the limit of quantification

PAGE S6 Non-target chemicals in u-cigarettes

PAGE S7 E-Cigarette products and summary of chemicals included in Figure 2 (PDF)

PAGE S10 JUUL flavors analyzed for aldehydes in Figure 4 (PDF)

<u>Table S1. List of Target Aldehydes and their Limits of Quantification</u>

#	Compound Name	Limit of Quantification (μg/mL)
1	Formaldehyde	10
2	Acetaldehyde	10
3	Acrolein	10
4	Propanal	10
5	Butanal	10
6	Crotonaldehyde	10
7	Pentanal	10
8	Glyceraldehyde	20
9	Dihydroxyacetone	20
10	5-Hydroxymethylfurfural	20
11	Glyoxal	10
12	Methylglyoxal	10

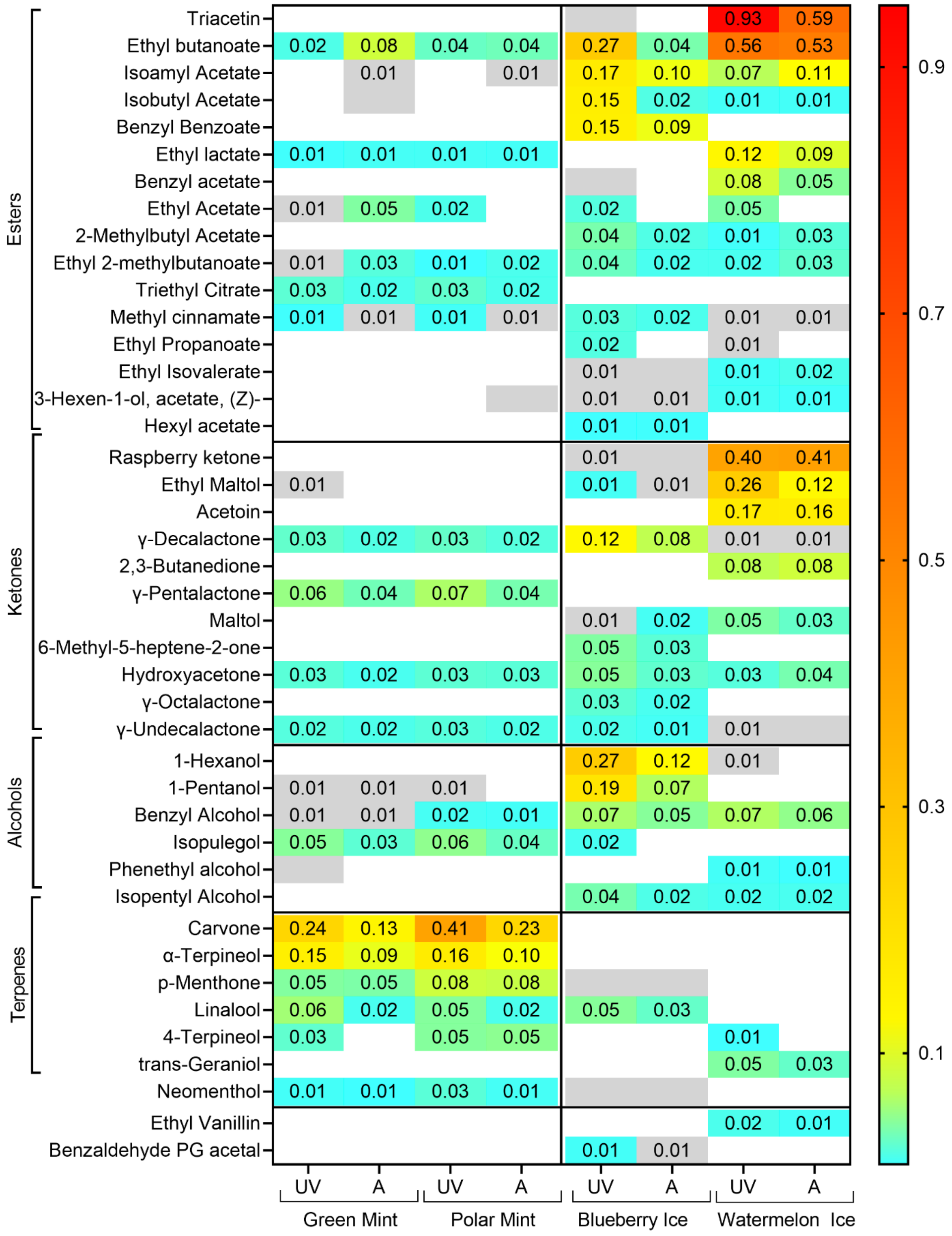

Figure S1. Heat map of flavor chemicals above the LOQ (0.02 mg/mL) with concentrations below 1 mg/mL in u-cigarettes. Chemicals are ordered on the *y*-axis based on chemical class and frequency of occurrence of flavor chemicals from top to bottom within each class. Products are grouped on the *x*-axis according to flavor type (mint vs ice). The color gradient on the right shows the concentrations of the flavor chemicals in the heat map. Grey spaces indicate concentrations <LOQ.

Table S2. Flavor Chemicals Below the Limit of Quantification ((10 μg/mL for Unvaped Fluids and 20 μg/mL for Aerosols)

		Green Mint		Polar Mint		Blueberry Ice		Watermelon Ice				
**Compound Name**	**CAS Number**	**UV**	**A**	**UV**	**A**	**UV**	**A**	**UV**	**A**	**Chemical Class**	**Hazards[1] Class**	**Odor Type**
Isoamyl Butyrate	106-27-4	4.6	13.1	5.4	13.3	ND	ND	2.1	4.4	Ester	Irritant	fruity
Menthyl Acetate	16409-45-3	3.2	5.7	6.5	8.7	2.5	4.8	8.9	11.8	Ester	ND	mentholic
Limonene	138-86-3	ND	9.4	ND	10.7	ND	ND	ND	ND	Terpene	Irritant	floral
Strawberry Glycidate_A	77-83-8	4.5	ND	ND	ND	6.1	0	9.1	7.1	Ester	ND	ND
Fenchol	1632-73-1	5.8	ND	9	ND	ND	ND	ND	ND	Terpene	Irritant	camphoreous
Amyl Acetate	628-63-7	3.4	6	3.3	ND	ND	ND	8.8	5.1	Ester	Irritant	fruity
β-Damascone	85949-43-5	5.1	4.4	8.2	5.2	7.5	4.6	5.1	4	Ketone	Irritant	fruity
Ethyl Cinnamate	103-36-6	ND	ND	ND	ND	ND	ND	8.2	5.9	Ester	Irritant	balsamic
2-Hexen-1-ol, (E)-	928-95-0	ND	ND	ND	ND	ND	ND	8.2	3.5	Alcohol	Irritant	fruity
α-Ionone	127-41-3	ND	ND	ND	ND	8.2	6.7	ND	ND	Ketone	Irritant	floral
Corylone	765-70-8	6.1	2.4	7.9	3.1	ND	ND	ND	ND	Ketone	Harmful	caramellic
Styralyl Acetate	93-92-5	3.6	ND	4.6	ND	7.8	7.4	ND	ND	Ester	ND	green
δ-Dodecalactone	713-95-1	ND	ND	ND	ND	ND	ND	6.2	5.5	Ketone	Irritant	tropical
Amyl Isovalerate	25415-62-7	ND	ND	ND	ND	ND	ND	7.5	ND	Ester	ND	fruity
2,3,5,6-Tetramethylpyrazine	1124-11-4	ND	ND	ND	ND	ND	ND	7.4	ND	Pyrazine	Harmful	nutty
Benzyl dimethylcarbinyl butyrate	10094-34-5	ND	ND	ND	ND	6.5	7	ND	ND	Ester	Irritant	floral
Acetylpyrazine	22047-25-2	4.6	ND	6.6	3.4	ND	ND	ND	ND	Pyrazine	Irritant	popcorn
Pulegone	89-82-7	4.5	ND	6.6	3.5	ND	ND	ND	ND	Terpene	Harmful	minty
(E)-β-Ionone	79-77-6	ND	ND	ND	ND	5.4	5	1.6	ND	Ketone	Irritant, D	floral
Piperitone	89-81-6	3.1	1.8	5.1	ND	ND	ND	ND	ND	Ketone	Irritant	herbal
2,3,5-Trimethylpyrazine	14667-55-1	4.4	ND	4.5	3.1	ND	ND	ND	ND	Pyrazine	Harmful	nutty
β-Pinene	127-91-3	ND	3.5	ND	4.3	ND	ND	ND	ND	Terpene	Harmful, D	herbal
Benzaldehyde	100-52-7	ND	ND	ND	ND	0.9	ND	4.1	3.1	Aldehyde	Harmful	fruity
Acetophenone	98-86-2	ND	ND	ND	ND	ND	ND	3.8	3.5	Ketone	Harmful	floral
Strawberry Glycidate_B	77-83-8	ND	ND	ND	ND	ND	ND	2.4	ND	Ester	ND	ND
α-Pinene	80-56-8	ND	2.5	ND	2.6	ND	ND	ND	ND	Terpene	Harmful, D	herbal
Guaiacol	90-05-1	ND	ND	ND	ND	ND	2	ND	ND	Phenol	Harmful	phenolic
Thymol	89-83-8	1	ND	ND	ND	ND	ND	ND	ND	Phenol	Corrosive, D	herbal

[1]D = Dangerous to the environment. ND = Not Determined. UV = unvaped. V = vaped.

Table S3. Non-target Chemicals in SURGE U-Cigarettes

Sample	Non-targets	Minor non-target
Blueberry Ice	1-ACETOXY-2-PROPANOL	Likely PG acetate ester isomer
	1,2-PROPANEDIOL, 2-ACETATE	
	Octamethyltetrasiloxane	Siloxane from silicone rubber etc.
	1,2,3-Propanetriol, monoacetate	GL acetate ester (monoacetin)
	Diacetin	GL acetate ester
	Raspberry ketone PG KETAL	
	2,2,4,4,6,6,8,8-Octamethyl-1,3,5,7,2,4,6,8-Tetraoxatetrasilocane	Siloxane from silicone rubber
Watermelon Ice	1-Acetoxy-2-Propanol	Likely PG acetate ester isomer
	Octamethylcyclotetrasiloxane	Siloxane from silicone rubber etc.
	2,6-Dimethylhept-5-Enal	
	Methyl Dihydrojasmonate	
	4-Octylbutan-4-Olide	
Green Mint	1-Acetoxy-2-Propanol	Likely PG acetate ester isomer
	2-Acetoxy-1-Propanol	Likely PG acetate ester isomer
	Octamethylcyclotetrasiloxane	Siloxane from silicone rubber
	1,2,3-Propanetriol, monoacetate	Likely GL acetate ester isomer
	(-)-Beta-Fenchol	
Polar Mint	1-Acetoxy-2-Propanol	Likely PG acetate ester isomer
	2-Acetoxy-1-Propanol	Likely PG acetate ester isomer
	Octamethylcyclotetrasiloxane	Siloxane from silicone rubber
	1,2,3-Propanetriol, monoacetate	GL acetate ester (monoacetin)

Acetate esters of propylene glycol and glycerol and siloxanes from silicon rubber were non-target chemicals estimated in u-cigarette aerosols.

Table S4. E-Cigarette Products and Summary of Chemicals included in Figure 2

Brand	E-liquid Flavor	Total Flavor Chemicals	WS-23	Nicotine	Reference.
SURGE	Green Mint	X	X	X	Current Study
SURGE	Polar Mint	X	X	X	Current Study
SURGE	Blueberry Ice	X	X	X	Current Study
SURGE	Watermelon Ice	X	X	X	Current Study
JUUL	Cool Mint	X		X	Omaiye et al 2019
JUUL	Cool Cucumber	X	X	X	Omaiye et al 2019
JUUL	Mango	X		X	Omaiye et al 2019
JUUL	Classic Menthol	X	X	X	Omaiye et al 2019
JUUL	Virginia Tob.	X		X	Omaiye et al 2019
JUUL	Classic Tob.	X		X	Omaiye et al 2019
JUUL	Fruit Medley	X		X	Omaiye et al 2019
JUUL	Crème Brulee	X		X	Omaiye et al 2019
PUFF	Sour Apple	X	X	X	Omaiye et al 2022
PUFF	Grape	X	X	X	Omaiye et al 2022
PUFF	Aloe Grape	X	X	X	Omaiye et al 2022
PUFF	Melon Ice	X	X	X	Omaiye et al 2022
PUFF	Lychee Ice	X	X	X	Omaiye et al 2022
PUFF	Mixed Berry	X	X	X	Omaiye et al 2022
PUFF	Clear (PP)	X	X	X	Omaiye et al 2022
PUFF	Cool Mint	X	X	X	Omaiye et al 2022
PUFF	Banana Ice	X	X	X	Omaiye et al 2022
PUFF	Tangerine Ice	X	X	X	Omaiye et al 2022
PUFF	Tobacco	X	X	X	Omaiye et al 2022
PUFF	Menthol	X	X	X	Omaiye et al 2022
PUFF	Café latte	X	X	X	Omaiye et al 2022
PUFF	Peach Ice	X	X	X	Omaiye et al 2022
PUFF	Cucumber	X	X	X	Omaiye et al 2022
PUFF	Pomegranate	X	X	X	Omaiye et al 2022
LIQUA	Two Apples US-KS1	X			Omaiye et al 2020
LIQUA	Mints US-KS	X	X		Omaiye et al 2020
LIQUA	Two Apple US-KS3	X			Omaiye et al 2020
LIQUA	Two Apple US-KS2	X			Omaiye et al 2020
LIQUA	Two ApplesNG-LG2	X			Omaiye et al 2020
LIQUA	Ry4 Tob. US-KS1	X		X	Omaiye et al 2020
LIQUA	Two Apples CN-GD	X			Omaiye et al 2020
LIQUA	Two ApplesNG-LG1	X			Omaiye et al 2020
LIQUA	Ry4 Tob. CN-GD	X		X	Omaiye et al 2020
LIQUA	Ry4 Tob. US-KS3	X		X	Omaiye et al 2020
LIQUA	Ry4 Tob.US-KS2	X		X	Omaiye et al 2020
LIQUA	Q Honeydew Drop US-KS	X			Omaiye et al 2020
LIQUA	Q Peach US-CA	X			Omaiye et al 2020
LIQUA	Q Peach UK-GB	X			Omaiye et al 2020
LIQUA	Peach US-CA	X			Omaiye et al 2020
LIQUA	Two Mints US-KS	X	X		Omaiye et al 2020
LIQUA	Q Peach US-KS	X			Omaiye et al 2020
LIQUA	Peach CN-GD	X			Omaiye et al 2020
LIQUA	Peach UK-GB	X			Omaiye et al 2020
LIQUA	Peach US-KS	X			Omaiye et al 2020

LIQUA	Q Pina Colada US-KS	X			Omaiye et al 2020
LIQUA	HP Sweet Accelerator US-KS	X			Omaiye et al 2020
LIQUA	Q Menthol (w Nic) US-KS	X			Omaiye et al 2020
LIQUA	Cheesecake CN-GD	X			Omaiye et al 2020
LIQUA	Q Menthol US-KS2	X			Omaiye et al 2020
LIQUA	Q Menthol US-CA	X			Omaiye et al 2020
LIQUA	Q Menthol US-KS1	X			Omaiye et al 2020
LIQUA	Energy Drink US-KS	X			Omaiye et al 2020
LIQUA	French Pipe Tob. US-KS	X			Omaiye et al 2020
LIQUA	Q Cherribakki US-KS	X			Omaiye et al 2020
LIQUA	HP Overdrive US-KS	X			Omaiye et al 2020
LIQUA	Coffee US-KS	X			Omaiye et al 2020
LIQUA	Coffee CN-XE	X			Omaiye et al 2020
LIQUA	Q Apple US-KS	X			Omaiye et al 2020
LIQUA	Apple US-KS2	X			Omaiye et al 2020
LIQUA	Coffee US-CA	X			Omaiye et al 2020
LIQUA	Q Apple UK-GB	X			Omaiye et al 2020
LIQUA	Q Apple US-CA	X			Omaiye et al 2020
LIQUA	Mild Kretek Tob. CN-GD	X			Omaiye et al 2020
LIQUA	Q Double Bubble US-KS	X			Omaiye et al 2020
LIQUA	Q Blackberry Jack US-KS	X			Omaiye et al 2020
LIQUA	Apple US-KS1	X			Omaiye et al 2020
LIQUA	HP Summer Drift US-KS	X			Omaiye et al 2020
LIQUA	Brownie CN-GD	X			Omaiye et al 2020
LIQUA	Licorice US-KS	X			Omaiye et al 2020
LIQUA	Licorice CN-GD	X			Omaiye et al 2020
LIQUA	Tiramisu CN-XE	X			Omaiye et al 2020
LIQUA	Q Fragola Fresca US-KS	X			Omaiye et al 2020
LIQUA	Tiramisu US-CA	X			Omaiye et al 2020
LIQUA	HP Fruity Velocity US-KS	X			Omaiye et al 2020
LIQUA	Banana US-KS	X			Omaiye et al 2020
LIQUA	Q The Moment US-KS	X			Omaiye et al 2020
LIQUA	Strawberry NG-LG	X			Omaiye et al 2020
LIQUA	Apple US-CA	X			Omaiye et al 2020
LIQUA	Apple CN-XE	X			Omaiye et al 2020
LIQUA	Apple UK-GB	X			Omaiye et al 2020
LIQUA	Cherry CN-XE	X			Omaiye et al 2020
LIQUA	Berry Mix CN-GD	X			Omaiye et al 2020
LIQUA	Cherry US-CA	X			Omaiye et al 2020
LIQUA	Q Piedmont Sunrise US-KS	X			Omaiye et al 2020
LIQUA	Q Berry Mix UK-GB	X			Omaiye et al 2020
LIQUA	Grape CN-XE	X			Omaiye et al 2020
LIQUA	Strawberry US-CA	X			Omaiye et al 2020
LIQUA	Q Berry Mix US-KS	X			Omaiye et al 2020
LIQUA	Vanilla NG-LG1	X			Omaiye et al 2020
LIQUA	Strawberry CN-XE	X			Omaiye et al 2020
LIQUA	Citrus Mix US-CA	X			Omaiye et al 2020
LIQUA	Citrus Mix CN-XE	X			Omaiye et al 2020
LIQUA	Citrus Mix US-KS	X			Omaiye et al 2020
LIQUA	Grape US-CA	X			Omaiye et al 2020
LIQUA	Vanilla NG-LG3	X			Omaiye et al 2020

LIQUA	Berry Mix US-CA	X			Omaiye et al 2020
LIQUA	Vanilla NG-LG2	X			Omaiye et al 2020
LIQUA	Vanilla NG-LG5	X			Omaiye et al 2020
LIQUA	Vanilla NG-LG7	X			Omaiye et al 2020
LIQUA	Vanilla NG-LG4	X			Omaiye et al 2020
LIQUA	Vanilla NG-LG6	X			Omaiye et al 2020
LIQUA	Berry Mix US-KS	X			Omaiye et al 2020
LIQUA	Vanilla US-KS	X			Omaiye et al 2020
LIQUA	Berry Mix CN-XE	X			Omaiye et al 2020
LIQUA	Q Golden Roanoke US-KS	X			Omaiye et al 2020
LIQUA	Virginia Tobacco CN-GD	X			Omaiye et al 2020
LIQUA	Cappucino US-KS	X			Omaiye et al 2020
LIQUA	Cola CN-GD	X			Omaiye et al 2020
LIQUA	Berry Mix UK-GB	X			Omaiye et al 2020
LIQUA	Chocolate US-KS	X			Omaiye et al 2020
LIQUA	Red Oriental Tob. CN-GD	X			Omaiye et al 2020
LIQUA	Red Oriental Tob. CN-GD	X			Omaiye et al 2020
LIQUA	Q Turkish Tob. US-KS	X			Omaiye et al 2020
LIQUA	Chocolate (w Nic) US-KS	X			Omaiye et al 2020
LIQUA	Cola US-KS	X			Omaiye et al 2020
LIQUA	Turkish Tobacco US-KS	X			Omaiye et al 2020
LIQUA	Vermillion Orient. Tob. CN-GD	X			Omaiye et al 2020
LIQUA	Blueberry US-KS	X			Omaiye et al 2020
LIQUA	Q Havana Libre US-KS	X			Omaiye et al 2020
LIQUA	Goldenrod Orient. Tob. CN-GD	X			Omaiye et al 2020
LIQUA	Cuban Cigar Tob. US-KS	X			Omaiye et al 2020
LIQUA	Golden Oriental Tob. CN-GD	X			Omaiye et al 2020
LIQUA	Menthol NG-LG1	X			Omaiye et al 2020
LIQUA	Menthol NG-LG2	X			Omaiye et al 2020
LIQUA	Menthol NG-LG3	X			Omaiye et al 2020
LIQUA	Menthol UK-GB	X			Omaiye et al 2020
LIQUA	Menthol US-KS	X			Omaiye et al 2020
LIQUA	Menthol CN-XE	X			Omaiye et al 2020
LIQUA	Menthol US-CA	X			Omaiye et al 2020
LIQUA	Watermelon US-KS2	X			Omaiye et al 2020
LIQUA	Watermelon US-KS1	X			Omaiye et al 2020
LIQUA	Bright Tob. CN-GD	X			Omaiye et al 2020
LIQUA	Bright Tob. US-CA	X			Omaiye et al 2020
LIQUA	Bright Tob. US-KS	X			Omaiye et al 2020
LIQUA	Bright Tob. UK-GB	X			Omaiye et al 2020
LIQUA	Bright Tob. CN-XE	X			Omaiye et al 2020

Table S5: JUUL Flavors Analyzed for Aldehydes in Figure 4

Country of Purchase	E-liquid Flavor
USA	Menthol 5%
USA	Menthol 3%
USA	Virginia Tobacco 5%
USA	Virginia Tobacco 3%
UK	Autunm Tobacco
UK	Blackcurrant Tobacco
UK	Virginia Tobacco
UK	Polar Menthol
UK	Ruby Menthol
UK	Summer Menthol
UK	Crispy Menthol
